# The Cotton States and International Exposition

**Published:** 1895-09

**Authors:** 


### THE COTTON STATES AND INTERNATIONAL EXPOSITION.

It is not often that we surrender space in these pages to matter entirely non-medical, but upon an occasion that means so much to this section of country as the Cotton States and International Exposition, which will hold the boards in Atlanta for the next three months, we depart from our usual rules, and give our readers notice that Atlanta has prepared a monster exhibition to instruct, entertain, and delight all who may come to see.

The *raison d'être* of this Exposition is the fact that at the late World's Fair, the South and her resources—agricultural, mineral, and manufacturing—were not justly and adequately represented. This enterprise will seek to make a full and complete exhibition of the industries of the Southern States, and to stimulate also the trade relations with Mexico and the South American republics.

The Exposition has a gratifying indorsement of the United States government. A handsome appropriation has been made and a magnificent building has been erected, in which each department of the government—War, State, Navy, Interior, etc.—will make a splendid exhibit. The exhibit of the Fish Commission will be one of the most complete of the government exhibits. Mexico, Venezuela, the Argentine Republic, Paraguay, Chili, Guatemala, Nicaragua, and other South American countries will make creditable exhibits. New York, Pennsylvania, Illinois, Tennessee, North Carolina, South Carolina, Florida, Louisiana, California, New Mexico, and other States will have suitable exhibits. There are thirteen principal exposition buildings: The Government, Manufactures and Liberal Arts, Fine Arts, Agriculture, Auditorium, Administration, Machinery, Fire, Minerals and Forestry, Transportation, Woman's, Electricity, and Negro. Among the musical features should be mentioned the engagement of the best musical organizations in the country: Gilmore's, Sousa's, and Innes's bands. Not the least among the attractions will be the reproduction of native villages, Mexican, Guatemalan, Eskimo, Japanese, Chinese, German, Indian, etc. The Vaudeville Theater, the Palace of Illusion, the Mystic Maze, the Scenic Railway, with Buffalo Bill's Wild West Show and Hagenbeck's Arena of Wild Animals, will furnish amusement for the thousands.

Come up to the Exposition. You can't afford to miss it. Get one of your medical brethren to look after your practice for a few days and come to Atlanta. Have your mail addressed in care of this JOURNAL, post-office box 431, and come in to get it at 330 Equitable Building.

